# Home-based cardio-oncology rehabilitation using a telerehabilitation platform in hematological cancer survivors: a feasibility study

**DOI:** 10.1186/s13102-023-00650-2

**Published:** 2023-03-23

**Authors:** Katerina Filakova, Andrea Janikova, Marian Felsoci, Filip Dosbaba, Jing Jing Su, Garyfallia Pepera, Ladislav Batalik

**Affiliations:** 1 Department of Rehabilitation, University Hospital Brno, Brno, Czech Republic; 2 Department of Rehabilitation and Sports Medicine, 2nd Faculty of Medicine, Charles University, University Hospital Motol, Prague, Czech Republic; 3 Department of Internal Medicine–Hematology and Oncology, University Hospital Brno, Brno, Czech Republic; 4 Department of Internal Medicine and Cardiology, University Hospital Brno, Brno, Czech Republic; 5 School of Nursing, The Hong Kong Polytechnic University, Hong Kong, China; 6 Clinical Exercise Physiology and Rehabilitation Research Laboratory, Physiotherapy Department, School of Health Sciences, University of Thessaly, Lamia, Greece; 7 Department of Public Health, Faculty of Medicine, Masaryk University, Brno, Czech Republic

**Keywords:** Cardio-oncology rehabilitation, Telerehabilitation, Home-based exercise, Cardiorespiratory fitness, Telemonitoring

## Abstract

**Purpose:**

Cardiovascular disease is a competing mortality cause in hematological cancer survivors due to toxic oncological treatment, accumulation of risk factors, and decline of cardiorespiratory fitness. Cardio-oncology rehabilitation (CORE) is an emerging treatment model to optimize the prognosis of hematological cancer patients and survivors; however, its accessibility during the COVID-19 pandemic is poor. The study aimed to evaluate the feasibility, safety, and effect of a 12-week home-based CORE intervention in telerehabilitation approach among hematological cancer survivors.

**Methods:**

A prospective single-arm interventional study was conducted at a faculty hospital in Brno, Czech Republic. This study provided 12 weeks of the home-based CORE using a telerehabilitation approach that allows remote supervision by a clinician from a medical facility. The telerehabilitation approach consists of three components: a heart rate sensor (PolarM430, Kempele, Finland), a web platform compatible with the sensor, and telesupervising via telephone call (1 call per week). To improve adherence, a physiotherapist called participants to assess or address adverse effects, exercise feedback, and participant-related concerns. The anthropometry, body composition, and cardiorespiratory fitness were measured immediately after the intervention.

**Results:**

Eleven hematological cancer survivors with an average age of 60.3 ± 10 years participated in the study. Most participants were diagnosed with Follicular lymphoma and received maintenance treatment. Participants had a significant (p < 0.05) increase in cardiorespiratory fitness by 2.6 ml/kg/min; and in peak workload, from 143.3 ± 60.6 W to 158.6 ± 67.5 W (p < 0.05). Improvement in anthropometry and body composition was observed but yielded no statistical significance. Most (80%) participants completed the three times/per week telesupervising exercise session for 12 weeks.No adverse event was identified.

**Conclusion:**

Findings from this study suggest that home-based CORE may provide hematological cancer survivors with an increase in CRF during the rehabilitation period after hospital discharge. The telerehabilitation CORE model is effective, feasible, safe, and has demonstrated good adherence. Further randomized controlled efficacy study with larger sample size is needed before clinical implementation.

**Clinical trial registration:**

Clinical trial registration number NCT04822389 (30/03/2021).

**Supplementary Information:**

The online version contains supplementary material available at 10.1186/s13102-023-00650-2.

## Introduction

Cardiovascular comorbidity among hematological cancer survivors could be leagthal [1]. The toxic effects of oncological treatment harm the cardiovascular system of patients and survivors during the acute phase of treatment and in the post-treatment period [2]. In addition, cancer survivors often experience an increased cardiovascular risk due to the accumulation of risk factors (hypertension, diabetes, dyslipidemia), frequently in combination with an unhealthy lifestyle (obesity, smoking, deconditioning) [3, 4]. Cardio-oncology rehabilitation (CORE) programs have been devised to reduce cardiovascular risk for hematological cancer survivors[5, 6]. The CORE model comprises a comprehensive prevention strategy based mainly on physical exercise.

Exercise should be an essential part of supportive cancer care, as it has been found to positively impact cardiovascular risk and a better prognosis after cancer diagnosis [7, 8]. Regular aerobic exercise increases cardiorespiratory fitness (CRF) and quality of life and reduces fatigue in hematological cancer survivors [9]. However, exercise programs are often underutilized due to logistical or time barriers. In addition, the coronavirus pandemic has further escalated this situation by limiting supportive care and mobility restrictions leading to increased sedentary behavior [10–12].

Telerehabilitation interventions represent promising alternatives that could bridge the participation and low utilization gap by facilitating home-based exercise. These telerehabilitation-based exercise interventions include walking or cycling, supported by telesupervising via telephone or text messages [13, 14]. However, the absence of exercise supervision in the home-based model may likely lead to safety concerns [15]. Recently, the development of information and communication technologies (ICT) has reduced the cost of services and the availability of the Internet. As a result, the chances of implementing telerehabilitation interventions increase. This medical care, which uses new ICT, is considered helpful for supporting home-based exercise interventions [16, 17].

Despite recently published research investigating the feasibility of exercise interventions delivered through telehealth for people affected by cancer, reporting improved outcomes and an overall positive experience [18, 19], there is a lack of data examining this approach from a cardiovascular prevention perspective in hematological cancer survivors. This article demonstrates a pilot study of a home-based CORE where a cardiac telerehabilitation model was integrated, enabling remote supervision by a clinician from a medical facility. The study aimed to evaluate the feasibility, safety, and effect of a 12-week home-based CORE intervention in cancer survivors.

## Material and research design

It was a prospective single-arm interventional study conducted at the University Hospital, Czech Republic. Participants enrolled between April 1, 2021, and May 31, 2022. The study followed the Declaration of Helsinki and Good clinical practice guidelines. Eligible participants were informed about the aims and purpose of the project and agreed to participate voluntarily by signing an informed consent form. The Ethics Committee of the University Hospital Brno approved the project (Brno, Czech Republic [no. 07-090621/EK]). The trial was registered in the ClinicalTrials.gov clinical trial registry under registration number NCT04822389 (30/03/2021). The study inclines to CONSORT guidelines of reporting pilot and feasibility trials conducted before a future definitive randomized controlled trial [20].

### Study sample

The study population was recruited from patients with Lymphoma managed and treated at the Department of Hematology and Oncology of University Hospital Brno. Potential patients were checked for eligibility by an oncologist based on their medical records. The inclusion condition for eligibility was (a) age between 18 and 80 years, (b) diagnosis of Lymphoma, c) ≤ 3 months after chemotherapy-based treatment (except ongoing adjuvant/maintenance biological treatment with anti-CD20 antibody), d) internet connection at home, e) information and communication technology skillfulness, f) signed informed consent.

The exclusion criteria were as follows: (a) inability to perform a cardiopulmonary exercise test (CPET), (b) severe psychological or cognitive disorders, (c) diagnosis with another active tumor or in a metastatic stage, (d) diagnosed heart and/or lung disease (recent occurrence of acute myocardial ischemia or chronic obstructive pulmonary disease within three months, severe aortic stenosis, end-stage heart failure, end-stage renal failure, and other systemic end-stage diseases), (e) planned intervention or operation, (f) participation in an exercise program during the previous six months, (g) severe immunosuppression or fever.

There was no prior information available for clinical trial design to base a sample size on. A sample size of 12 participants per group was recommended for such pilot studies. The justifications for this sample size are based on rationale from a study by Julious SA [21].

### Data collection

Home-based CORE was conducted by a qualified physiotherapist who was not involved in the assessments. The cardiologist performed CPET clinical assessment at baseline and after the intervention. This cardiologist was blinded to the stage of CPET. Study data were encoded in a spreadsheet and sent to a statistician to perform the data analysis. At the end of each assessment, the data was stored, and the researchers had no further access.

## Outcome measures

### Anthropometry and body composition assessment

Height was measured with a stadiometer with 0.1 cm precision (KERN MPE 250 K, Großmaischeid, Germany), while weight was measured with a portable scale with a precision of 0.1 kg (SECA 861®, SECA, Hamburg, Germany). Body composition was assessed by bioelectrical impedance analysis using the multifrequency analyzer (InBody 370 S, Seoul, Korea). According to the manufacturer’s instructions, each participant remained in contact with the hand and foot electrodes of the device. At the same time, 15 measurements were taken using three different electrical frequencies (5 kHz, 50 kHz, 250 kHz) through each of the five touch segments of the participant’s right arm, left arm, torso, right leg and left leg. The device then measured the impedance of electrical currents to evaluate several indicators of body composition, including body fat mass, visceral fat, skeletal muscle mass, extracellular water, intracellular water, and total body water. The entire bioelectrical impedance analysis assessment carried one minute to complete.

### Cardiopulmonary exercise test

Determination of maximal HR was achieved using a progressive incremental CPET on an Ergoselect 100 bicycle ergometer (Ergoline, Bitz, Germany) up to the symptom-limited maximum of each participant. CPET was completed following the European Society of Cardiology guidelines and the American Cardiology Association [22]. The role of CPET in clinical cardiology lies mainly in the means of diagnosis and prognosis as well as exercise training [23]. The peak and resting HR assessments with a standard 12-lead electrocardiogram, gas exchange, and blood pressure were continuously recorded and estimated through CPET. Blood pressure was measured manually with a CA-MI tonometer (CAMI Group, Parma, Italy) every 2 min. A study physician supervised the testing in case of an adverse event or occurrence of complications.

### Participant flow

After enrollment, eligible participants were instructed on how to use telerehabilitation devices. All participants underwent baseline examination with echocardiography, CPET, and bioimpedance analysis. CPET and bioimpedance analysis were repeated at the end of the intervention (12 weeks follow-up). Adherence to the training prescription (adherence to exercise intensity and duration) was monitored using a training diary, and adverse events associated with exercise were recorded. Adherence to the intervention was considered valid when at least 70% of the prescribed exercise sessions were completed. At the end of the intervention, the participants evaluated the applicability of the telerehabilitation intervention using a Visual analog scale (0–10). The resulting assessment was determined, 0–3 points for low satisfaction, 4–7 points for average satisfaction, and 8–10 points for high satisfaction.

**Fig. 1:**
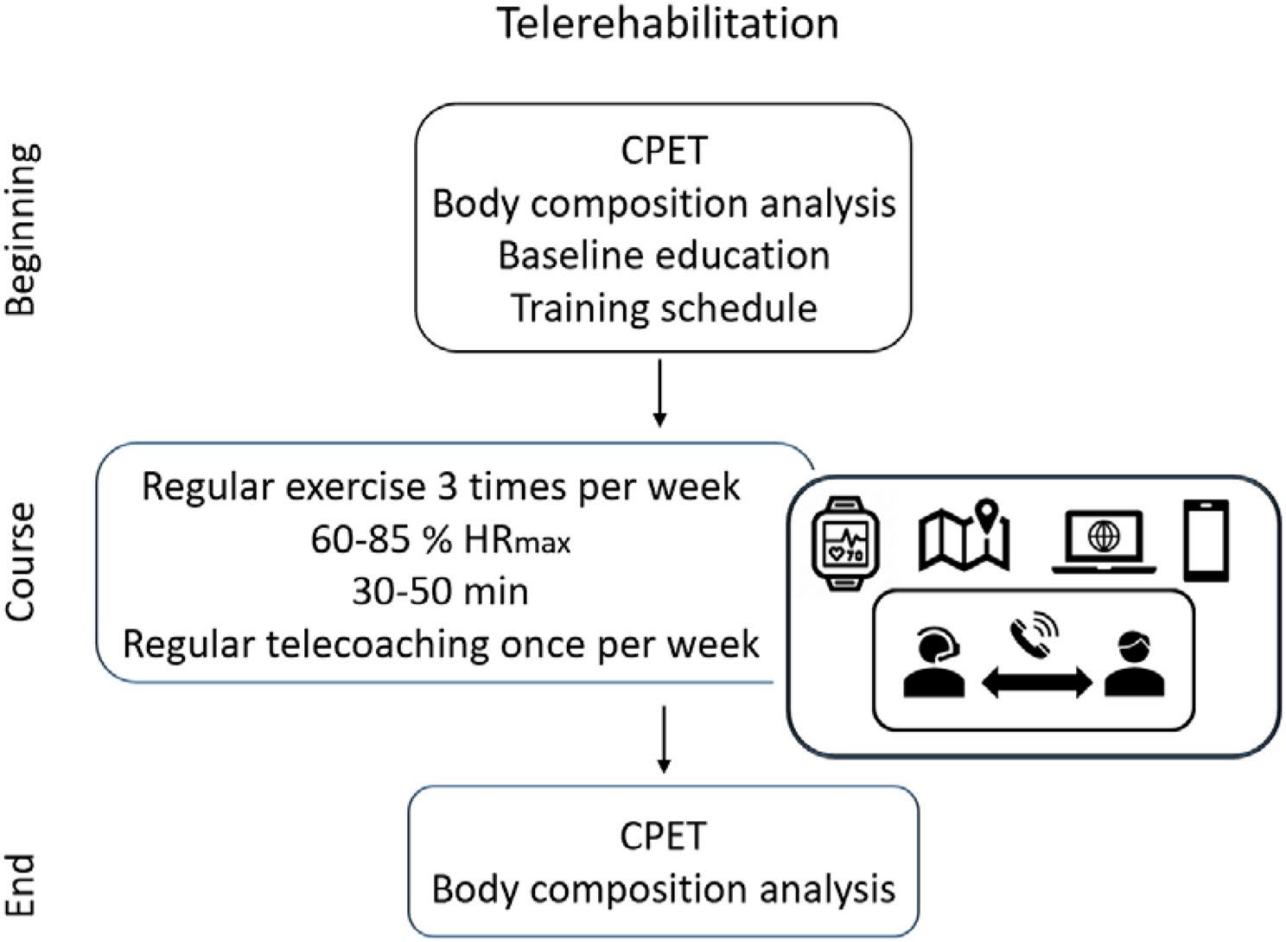
Scheme of rehabilitation approach

### Intervention

**Fig. 2:**
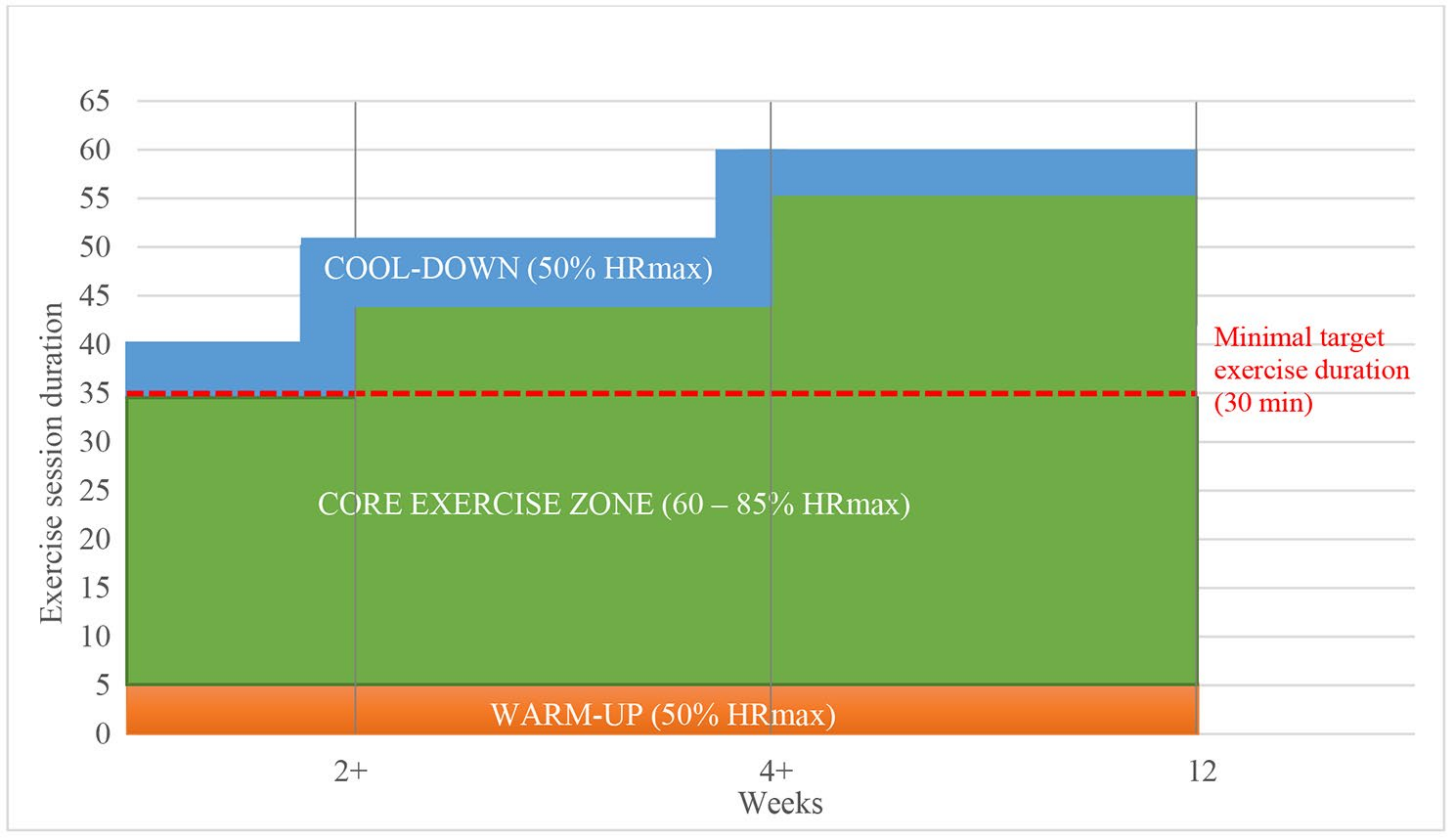
Gradual 12-week exercise prescription

The intervention (Table 1) of this study was 12 weeks of home-based CORE (3 sessions per week) using a telerehabilitation approach that allows remote supervision by a clinician from a medical facility (Fig. 1). The telerehabilitation approach consists of three components: a HR sensor (PolarM430, Kempele, Finland), a web platform (PolarFlow, Kempele, Finland) compatible with the sensor, and telesupervising via telephone call (1 call per week). The telerehabilitation components were loaned to the participants along with a manual for the sensor and the web platform use. Moreover, participants obtained a personal questionnaire (sex, age, diagnosis, and pharmacological treatment), a trial manual, and an educational booklet (nutrition advice, obesity management, diabetes mellitus management, smoking cessation, strength, and flexibility exercise examples). The HR sensor instruction lasted approximately 30 min during the baseline assessment. Additional 60 min were used to conduct a practice exercise session to familiarize them with data uploading and review the web-based training diary. The HR training zone was determined based on baseline CPET (60–85% HRmax) and Rating of Perceived Exertion (11–13 degrees on a 1–20 scale) according to recommendations [24]. The training session duration was prescribed gradually (Fig. 2). The training modality was determined according to the participant´s preference. Modality of walking, Nordic walking, or cycling was recommended.

**Table 1:** Detailed outline of the intervention according to the TiDier template

Item No	Item
**Brief name**	
1	Exercise-based CORE telerehabilitation intervention to increase cardiorespiratory fitness
**Why**	
2	Remote supervision of exercise-based rehabilitation may result in a more individualised approach, increased patient responsibility, and enhanced compliance, which may lead to improvement in the cardiovascular disease prevention
**What**	
3	Participants received a telemonitored exercise training programe with telesupervision guidance through a web-based platform and telephone call.
4	The exercise prescription was self-monitored by the participant using a HR sensor (Polar M430) with a gradual increase in exercise intensity.
**Who provided**	
5	A physiotherapist specializing in cardiac rehabilitation for more than five years has provided CORE telerehabilitation intervention to participants individually.
**How**	
6	The physiotherapist, via a web-based platform (PolarFlow), telemonitored and telesupervised participants post-exercise and telephone telesupervision was provided once a week. From weeks 0 to 12, participants received regular weekly phone calls (10 to 20 min) to monitor for adverse effects, promote compliance and adherence to the study protocol, address any participant questions or concerns, and gather information about participants’ current symptomatology.
**Where**	
7	Participants were recruited from a municipal oncology clinic that provides care to patients and survivors from the entire South Moravian region of the Czechia
**When and How Much**	
8	Participants were encouraged to exercise three times a week for 12 weeks at a level of 60 to 85% HRmax and 11 to 13 on the Borg rating of RPE
**Tailoring**	
9	Participants began with 30 min of exercise in the first two weeks and then the duration increased up to 50 min (Fig. 2).
**Modifications**	
10*	NA
**How well**	
11	Adherence to the training prescription was monitored using a web-based training diary. Adherence to the intervention was considered valid when at least 70% of the prescribed exercise sessions were completed. At the end of the intervention, the participants evaluated the applicability of the telerehabilitation intervention using a Visual analog scale (0–10). The resulting assessment was determined, 0–3 points for low satisfaction, 4–7 points for average satisfaction, and 8–10 points for high satisfaction.
12*	The mean participation rate in the planned exercise sessions was 78.2%. On average, the participant completed 30.5 ± 6.8 sessions (range: 13–36). The average time of one training session was 43.9 ± 11.6 min. Participants maintained the training intensity at 79.7 ± 4.1% of HRmax.

A physiotherapist specializing in cardiac rehabilitation for more than five years has individually provided home-based CORE intervention to participants. The physiotherapist, via the telerehabilitation platform, telemonitored and telesupervised participants post-exercise. Data from each training session (HR, training duration) were recorded during telemonitoring. From weeks 0 to 12, participants received regular weekly phone calls (10 to 20 min) to monitor for adverse effects, promote compliance and adherence to the study protocol, address any participant questions or concerns, and gather information about participants’ current symptomatology. Telephone counseling was scheduled for a specific day and time each week of the intervention. Through a web-based training diary, the study physiotherapist supervised the training sessions of the participants and analyzed the training data. A web-based training diary (PolarFlow, Kempele, Finland) uses the Internet to connect a wearable sensor to display recorded electronic health data.

### Statistical analyses

Study data were expressed using statistical descriptions, such as means and standard deviations for continuous variables and numerical variables. Continuous variables were compared between study groups using a T-test and numerical variables using the chi-squared test. A two-tailed Mann–Whitney U-test was performed for collected data that had not been normally distributed. The statistical significance level was set at p < 0.05 (two-tailed) for all differences between study groups. All study data were processed and analyzed in computerized statistical software Statistica 12 (TIBCO, Software Inc., Palo Alto, CA, USA).

**Fig. 3:**
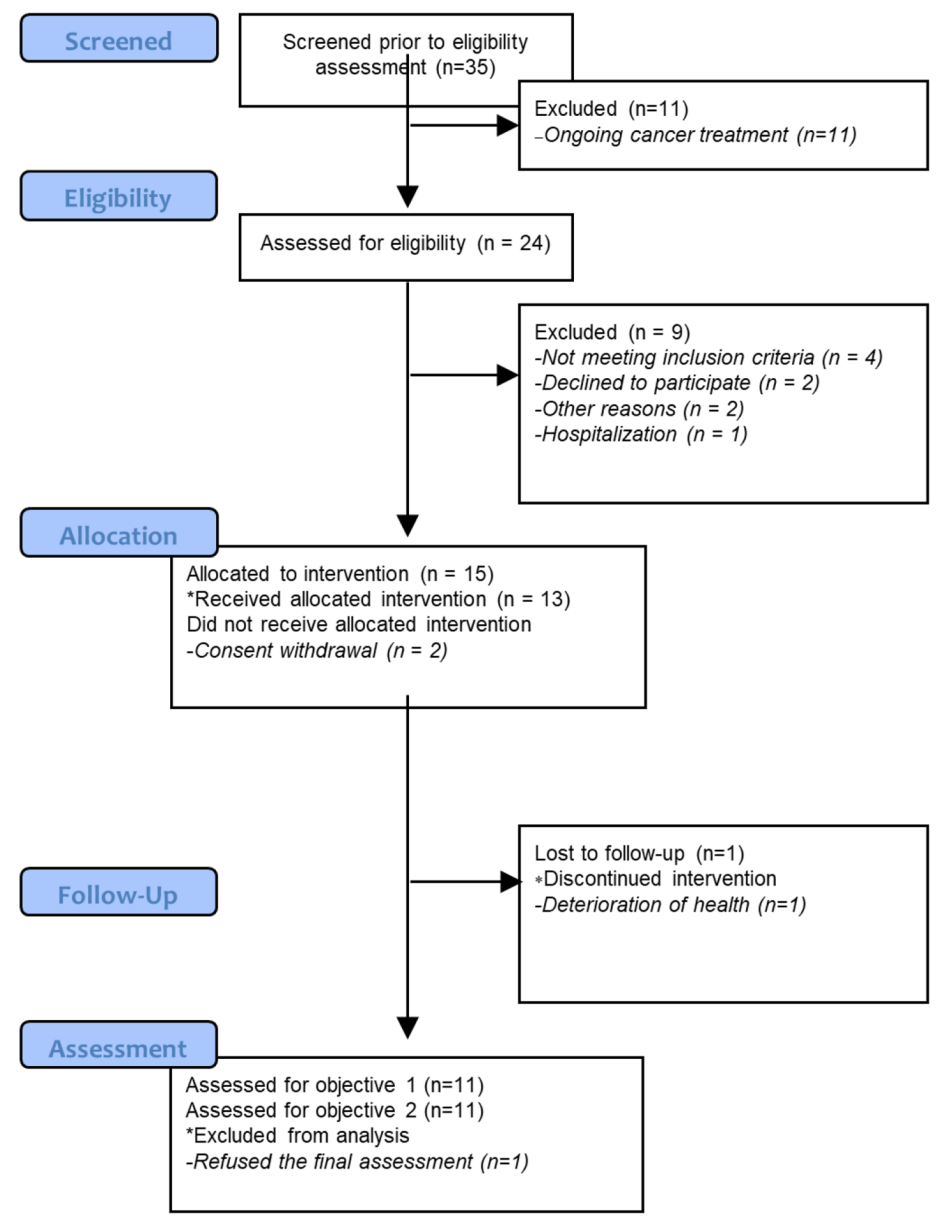
Flow chart of the study

## Results

Twenty-four participants were prospectively invited to the study, of whom nine were excluded. A total of fifteen participants (62.5%) were included in this study, and twelve (80%) completed the intervention. A more detailed flow of the study is shown in Fig. 3. Table 2 shows the baseline demographic and clinical characteristics of the participants. The average age was 60.3 ± 10 years (range 33–72 years), and 73% were women. Most participants were diagnosed with Follicular Lymphoma and received rituximab maintenance (anti-CD20 antibody) treatment. Participants had preserved left ventricular function before intervention (mean, 60.9 ± 6.1%).

**Table 2:** Characteristics of the participants

Variable	Home-based CORE (n = 11)
Age [M (SD)]	60.3 (10.2)
< 60 years (n)	2 (18%)
≥ 60 years (n)	9 (82%)
Sex	
Female (n)	8 (73%)
Male (n)	3 (27%)
Body mass index [M (SD)]	28.3 (3.0)
Average weight (n)	3 (27%)
Overweight and Obese (n)	8 (73%)
Marital status	
Not married (n)	3 (27%)
Married (n)	8 (73%)
Children living at home	
None (n)	8 (73%)
One or more (n)	3 (27%)
Education	
University not completed (n)	6 (55%)
Completed university (n)	5 (45%)
Employment status	
Not retired (n)	6 (55%)
Retired (n)	5 (45%)
Cancer type	
Folicular lymphoma (n)	8 (73%)
Diffuse large B-cell lymphoma (n)	3 (27%)
Years since diagnosis [M (SD)]	1.6 (1.3)
≤ 3 years (n)	9 (82%)
> 3 years (n)	2 (18%)
Current cancer status	
Disease free (n)	8 (73%)
Existing disease (n)	3 (27%)
Treatment status	
Receiving maintenance treatment (n)	8 (73%)
Completed treatments (n)	3 (27%)
Stage at diagnosis	
Stage II (n)	4 (36%)
Stage III (n)	4 (36%)
Stage IV (n)	3 (28%)
Cardiovascular characteristics	
Diabetes mellitus (n)	3 (28%)
Hypertension (n)	5 (45%)
Dyslipidemia, (n)	4 (36%)

maintenance treatment - rituximab 1.400 mg, subcutaneous injections (every 3 months, up to 2 years);

The primary results are shown in Table 3. After completing the 12-week home-based CORE, participants showed a significant (p < 0.05) increase in CRF by 2.6 ml/kg/min, with statistical power of 0.43 (Fig. 4). Peak workload also significantly improved, from 143.3 ± 60.6 W to 158.6 ± 67.5 W (p < 0.05). Lean mass only increased from 31.5 ± 8.8 kg to 31.7 ± 8.4 kg, while the change was insignificant. Fat mass decreased from 27.0 ± 7.6 kg to 26.6 ± 7.9 kg; these differences also did not reach statistical significance.

**Table 3:** Study results

Variable	Mean	SD	95% [CI]
Baseline pVO_2_ (ml/kg/min)	20.6	7.0	[15.89, 25.30]
12-week pVO_2_ (ml/kg/min)	23.2	8.5	[17.49, 28.91]
Difference	-2.6	2.5	[-4.32, 9.52]
Baseline pW (watt)	143.3	60.6	[102.58, 184.01]
12-week pW (watt)	158.6	67.5	[113.25, 203.94]
Difference	-15.4	17.0	[-41.75, 72.35]
Baseline pRER (value)	1.22	0.1	[1.15, 1.28]
12-week pRER (value)	1.22	0.1	[1.15, 1.28]
Difference	0	0.06	[-0.08, 0.08]
Baseline HRmax (beats/min)	144.6	20.0	[131.16, 158.03]
12-week HRmax(beats/min)	141.0	18.8	[128.37, 153.63]
Difference	3.6	6.0	[-13.66, 20.86]
Baseline BMI (kg/m^2^)	28.3	3.0	[26.28, 30.31]
12-week BMI (kg/m^2^)	28.3	3.2	[26.15, 30.45]
Difference	0	0.4	[-2.75, 2.75]
Baseline Fat mass (kg)	27.0	7.6	[21.89, 32.11]
12-week Fat mass (kg)	26.6	7.9	[21.29, 31.90]
Difference	0.4	1.1	[-6.49, 7.29]
Baseline Lean mass (kg)	31.5	8.8	[25.58, 37.41]
12-week Lean mass (kg)	31.7	8.4	[26.05, 37.34]
Difference	-0.2	0.5	[-7.45, 7.85]

SD, standard deviation; RER, respiratory exchange ratio; HR, heart rate; ES effect size, CI confidence interval, BMI, body mass index; pVO_2,_ peak oxygen consumption.

All 11 participants successfully accessed the web-based platform, registered for exercise training, and remained active during the 12-week intervention. The rate of participation in the planned training sessions was 78.2%. On average, the participant completed 30.5 ± 6.8 sessions (range: 13–36). The average time of one training session was 43.9 ± 11.6 min. Participants maintained the training intensity at 79.7 ± 4.1% of HRmax. In 35 cases out of 366 (9.6%, range 1–7), target training intensity was not achieved, and telesupervising was needed. 4 out of 11 (37%) participants followed the training prescription without deviations. In 13 cases out of 366 (3.6%) training sessions, data was lost due to improper handling of the HR sensor or a discharged battery. The mean rate of participation in telesupervising was 84.2%. On average, the participant completed 10 of the 12 planned phone calls.

Regarding safety, no serious adverse events leading to unplanned hospitalization were reported, and no participant made an emergency call during the study. Mild effects leading to dropout from planned sessions were fatigue, colds, and muscle pain in the limbs, and were observed in 21 cases. These effects leading to cancellation exercise were subsequently addressed by telesupervising, where it was recommended to reduce the subsequent training time by 5 to 10 min and moderate the intensity to the lower HRmax training level. Participants were generally satisfied with the applicability of the telerehabilitation approach (visual analog scale mean value, 7.3 ± 2.1). The physiotherapist and cardiologist involved in the telemonitoring did not experience significant data quality or transmission problems.

**Fig. 4:**
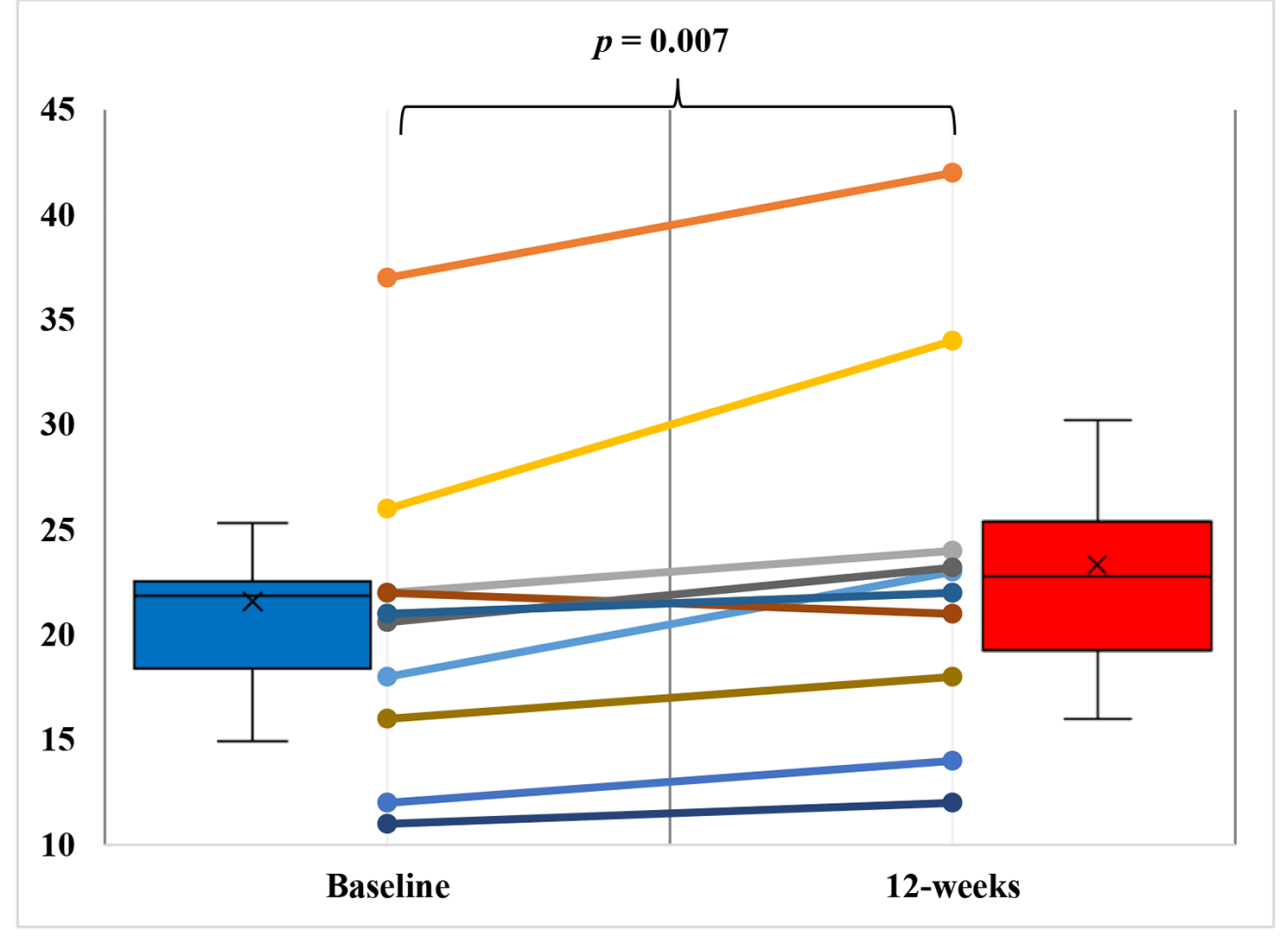
The change in peak oxygen uptake from baseline to 12 weeks. Box plots indicate peak oxygen uptake values for the whole cohort (n = 11). Line plots indicate changes for individual participants

## Discussion

This study investigates the feasibility, safety, and effect of a 12-week home-based CORE intervention using a telerehabilitation approach in hematological cancer survivors,. There were three primary findings. First, CRF assessed by peak oxygen uptake increased significantly due to exercise intervention. Second, the high rate of successful completion of the home-based CORE using a combination of telerehabilitation and telesupervising. The participation rate in the three times week exercise remained at almost 80% after 12 weeks. Third, there were no training-related severe adverse events during the intervention.

Integrating the exercise-based cardiac rehabilitation model as a form of CORE seems promising as it may reduce in cardiovascular risk and improve prognosis after a cancer diagnosis. The REACT study used center-based exercise therapy in cancer survivors who completed 36 sessions over 12 weeks, and participation was 67% [25]. In our current study, participants completed the 12-week home-based CORE intervention with a higher participation rate of 78.2%. The result is crucial because low adherence and participation levels cannot achieve an exercise intervention’s expected health benefits. Additionally, our participation rate is similar to the rate reported in a systematic review of remotely monitored cardiac telerehabilitation interventions [26, 27]. As identified in the study by Hardcastle et al., difficult access and availability of center-based exercise programs are the main barriers to non-participation [28]. Home-based CORE using a telerehabilitation approach such as our intervention has the potential to overcome the physical, psychosocial and environmental barriers faced by center-based exercise models [29–31]. However, increasing patient participation and adherence to exercise interventions may require individualized management.

Alternative strategies have achieved additional significant attention in the recent era associated with the coronavirus pandemic, with frequent quarantine and restrictions limiting standard care [32]. Several studies have investigated the home-based exercise approach in cancer survivors. Sagarra-Romero et al. evaluated the usefulness of home-based indoor exercise using a computer, web-based telecommunication applications, and a wearable HR monitor [33]. Larkin et al. investigated the effectiveness of a virtual home-based program for breast cancer survivors provided through educational seminars via an online platform in combination with telephone calls and/or text messages [34]. Alibhai et al. evaluated the feasibility and usefulness of home-based exercise training in prostate cancer using teleconsultations [35]. This telehealth evidence suggests that an alternative approach is feasible and provides physiological and psychological benefits for cancer patients and survivors even during a pandemic. There is, therefore, a reasonable assumption that telehealth can represent a practical alternative approach to supportive oncological care in the future.

Safety is crucial in home-based exercise interventions with remote guidance from the center. Regarding the safety of home-based cardiac rehabilitation, the latest study from 2022 revealed an incidence of 1 severe adverse event per 23.823 participant-hours of home-based exercise in a sample of 808 participants [15]. It should be noted that no life-threatening side effects were observed in the review, and more than half of the sample were participants in high-risk stratification. A recent review of cardiac telerehabilitation also demonstrated that no serious adverse events or deaths were recorded during the exercise intervention [26]. In our current study, although several physical problems occurred, no participant reported an emergency, and no serious adverse events were reported. These observations suggest that our telerehabilitation platform can be safely used in the home-based CORE model, even in elderly participants.

The above studies included participants on average 50–60 years old; compared to our home-based CORE intervention, the average age was 60. Although there is an assumption that older participants in telerehabilitation interventions need more technological skills, our results showed that a remotely guided approach could also be applied to relatively old persons. The concept of our telerehabilitation approach is based on the replication of the characteristics of center-based cardiac rehabilitation in a home-based setting. One of the strengths of our approach is the provision of telesupervising to the participant, which can facilitate and encourage participation in the program’s exercise routines. These aspects contributed to increased adherence. Further studies are needed to elucidate the optimal approach to maximize the effectiveness of home-based CORE.

Physical fitness is an essential predictor of total mortality [36]. Current evidence on the prognostic impact of CRF supports the clinical relevance of developing effective strategies to improve CRF levels in cancer patients and survivors [6, 37]. In this study, we found a significant improvement in CRF after the intervention. The approximately 13% increase in maximal oxygen uptake was comparable to previous studies on the effectiveness of exercise-based interventions in cancer survivors [38]. A systematic review of six studies showed a significant improvement in CRF after home-based exercise intervention compared to a control group [26]. Previous controlled exercise studies have confirmed the effectiveness of aerobic interventions in a wide range of cancer patients and survivors [39, 40]. Physically fit survivors have about a 37% lower risk of dying from cancer than survivors who exercised the least [41]. It has been found that higher levels of CRF may be associated with better improvement in adherence, exercise prescription, and fatigue levels [25]. Therefore, the effect of HB exercise interventions in the context of CRF may be crucial. Training intensity was determined using baseline CPET and then prescribed according to HR zone (60–85% HRmax). Participants showed individually maintained an average intensity of 79.7% HRmax during exercise. Aerobic exercise is a high-fidelity and recommended intervention to increase CRF [42]. Fidelity is one of the crucial implementation outcomes to understand when introducing new interventions to maintain the quality of the intervention [43]. Studies suggest that adherence to cardiac telerehabilitation can be at least as high as center-based programs [26, 27, 44]. However, when prescribing exercise, it is essential to consider that CRF results vary if chemotherapy is given in different doses [38]. Therefore, CORE exercise specialists should understand chemotherapy regimens since they might lead to different training outcomes [45].

Accurate body composition and muscle-mass preservation assessment can reduce chemotherapy’s toxicity and improve overall survival [46]. In this study, we found no improvement in body composition, in contrast to a systematic review of home-based exercise interventions in cancer survivors where significant changes in body composition were noted [16]. The most likely reason for the non-significant body composition results may be a lack of power due to the small sample size, also acknowledging that body composition was not the primary endpoint of this study. Including an aerobic component and only educational strength training recommendations are probably insufficient. This limitation could be solved by adjusting the exercise prescription of individualized strength training with one-arm weights or resistance bands. According to the systematic review by Nascimento et al., the inclusion of muscle-strengthening and aerobic activities is also crucial because they may reduce total cancer mortality [47].

Training intensity was determined by baseline CPET and then adjusted according to the HR zone (60–85% HRmax), and during the exercise session, the participants individually maintained an average intensity of 79.7% HRmax. No increase in exercise volume was observed during the intervention. Since this was the first pilot study using home-based CORE, the workload was not intensively escalated from a safety point of view.

Several limitations of this study need to be mentioned. Participants involved in the study may have been more motivated than the general population of cancer survivors. There may also have been some degree of selection bias. Because this was a single-arm study with a small sample size, we cannot conclude that home-based CORE participation rates using the telerehabilitation model are higher than usual care. A more extensive study designed as an RCT could provide the answer [48, 49]. It should also be mentioned that several ICT and mechanical problems led to the cancellation or interruption of exercises during the study (3.6% of sessions), subsequently related to the loss of training data. Most of the lost data was related to battery charging or handling issues. Likely, further ICT development and availability of innovative ICT charging options, specifically the wireless charging pad and increasing technological literacy of the population, can alleviate these limitations [50]. In addition, other essential action points of the telehealth and ICT issue for practical implications are solutions for data integration in electronic medical records and their security and privacy.

Finally, although the study demonstrated the feasibility of adopting new alternative strategies for cancer survivors, the findings should be interpreted cautiously. The small sample of most patients with hematological malignancies limits the interpretation of the results. This preliminary evidence for the application of home-based CORE encourages further research to conduct a larger study to detect a true effect and test this population’s long-term exercise efficacy.

## Conclusion

Participation rates in exercise-based cancer rehabilitation are a well-documented and persistent problem. Addressing this challenge is limited research that has utilized alternative strategies based on remotely conducted home-based methodology, as outlined in recent reviews. Incorporating CORE recommendations with telerehabilitation revealed new preliminary findings for cancer survivors. Results from this study suggest that home-based CORE may provide hematological cancer survivors with an increase in CRF during the rehabilitation period after hospital discharge. Given the combined effects of cancer treatment that increase the risk of morbidity and mortality, there is good reason to identify at-risk groups of patients and survivors who provide individualized exercise interventions, particularly those with barriers to accessing centralized services. Further randomized controlled efficacy study with larger sample size is needed before clinical implementation.

## Electronic supplementary material

Below is the link to the electronic supplementary material.

Supplementary Material 1

## Data Availability

The datasets generated and analysed during the current study are available from the corresponding author upon reasonable request.
